# Understanding urine output in critically ill patients

**DOI:** 10.1186/2110-5820-1-13

**Published:** 2011-05-24

**Authors:** Matthieu Legrand, Didier Payen

**Affiliations:** 1 Department of Anesthesiology and Critical Care and SAMU, Lariboisière Hospital, Assistance Publique- Hopitaux de Paris; University of Paris 7 Denis Diderot, 2 rue Ambroise-Paré, 75475 Paris Cedex 10, France

## Abstract

Urine output often is used as a marker of acute kidney injury but also to guide fluid resuscitation in critically ill patients. Although decrease of urine output may be associated to a decrease of glomerular filtration rate due to decrease of renal blood flow or renal perfusion pressure, neurohormonal factors and functional changes may influence diuresis and natriuresis in critically ill patients. The purpose of this review is to discuss the mechanisms of diuresis regulation, which may help to interpret the urine output in critically ill patients and the appropriate treatment to be initiated in case of changes in urine output.

## Introduction

Acute renal failure or acute kidney injury (AKI) is defined by an acute decline of glomerular filtration rate (GFR). Occurrence of AKI is associated with substantial in-hospital mortality, exceeding 50% when AKI is part of a multiple organ failure syndrome [1,2]. Therefore, early recognition of AKI, better understanding of its pathogenesis, and development of preventing strategies appear to be potential areas of improvement of patient's prognosis. The decrease of glomerular filtration rate and urine output in response to a decrease of renal blood flow is classically referred as pre-renal azotemia, which can evolve into structural damage if renal hypoperfusion persists. In this line, urine output often is used as a marker of AKI but also to guide fluid resuscitation in critically ill patients. However, both the contribution of renal hypoperfusion to AKI and the genuine definition of pre-renal and intra-renal azotemia have been challenged by several authors [3-5]. The recent international consensus conference on acute renal failure therefore recommended the term "acute kidney insufficiency" rather than "acute kidney injury" in the light of paucity of evidence of a relation between tissue damage and organ failure in human AKI [6]. The purpose of this review is to discuss the mechanism of diuresis regulation and the interpretation of urine output in critically ill patients in the light of clinical and physiological studies.

## Why should we wonder about oliguria and AKI?

There is accumulating evidence that critically ill patients developing AKI have an increase relative risk of death. Occurrence of AKI is a marker of severity of the underlying acute illness but also appears as an independent factor associated with mortality in unselected critically ill patients [7], in sepsis [8], pneumonia [9], or cardiac surgery [10]. The mechanistic pathways of such an association remain elusive, with intrication of inflammation, metabolism, and apoptotic phenomena. Remote organs damage has been suggested in several experimental studies [11,12]. Ischemic-induced AKI has been found to induce myocardial apoptosis [13], to activate lung inflammatory and apoptotic pathways, and to increase lung water permeability [14]. Surprisingly, even a small increase of serum creatinine after cardiac surgery or transient (i.e., reversible within 3 days) AKI has been found to be associated with an increased risk of death [15]. Although fluid resuscitation and optimization of renal perfusion pressure are central to the prevention and treatment of AKI, excessive fluid resuscitation may be harmful in some critically ill patients. Payen et al. [16] and Bouchard et al. [17] found, when analyzing two large cohorts of critically ill patients, that a positive fluid balance was associated with an increased risk of death in patients suffering from AKI. First, aggressive fluid resuscitation, although increasing renal blood flow, can be ineffective in restoring renal microvascular oxygenation due to hemodilution with no increase in blood-oxygen carriage capacities [18]. Second, positive fluid balance can deteriorate cell oxygenation and prolong mechanical ventilation [19]. Finally, fluid overload may lead to central venous congestion and decrease of renal perfusion pressure [20], which will promote the development of AKI in patients with acute heart failure [21] or sepsis [22]. The type of fluid used also can have a role with "renal toxicity" associated with the use of colloids.

## Urine output and definition of acute kidney injury

In clinical research, more than 30 definitions of acute renal failure have been used before the release of the RIFLE criteria by the Acute Dialysis Quality Initiative group in 2004 [23]. The first merit of this classification was to introduce a standard and simple definition of AKI for clinical research purposes but also to stratify the severity of AKI based on serum creatinine level, creatinine clearance, or urine output. In 2007, the Acute Kidney Injury Network classification was published, introducing subtle modifications to the RIFLE criteria. A part from the change in nomenclature (Risk, Injury, and Failure were replaced by stage 1, 2, and 3, the categories Loss and Endstage disappeared), an absolute increase of serum creatinine of 0.3 mg/dl was sufficient to classify patients in stage 1, introducing the notion than only small changes in serum creatinine are of clinical relevance. Finally, the AKIN criteria should be applied "after following adequate resuscitation when applicable" with the purpose of excluding patients with pure renal pre-azotemia. The introduction of the RIFLE and AKIN definitions were a crucial step forward in the development of clinical research and have since been widely accepted by the medical community. Using these classifications, a patient with decrease of urine output will be classified as "AKI." However, a nonsustained decrease of urine output does not necessarily imply a decrease of glomerular filtration rate but can simply represent a physiological renal adaptation (i.e., antidiuresis and antinatriuresis) to maintain the body volume and/or electrolytes homeostasis. This would be the case if decreased urine output is not associated with a decline of creatinine clearance. Although severe acute renal failure with oliguria or anuria has been reported to be associated with a worse outcome compared with patients with preserved urine output, the use of urine output as a criterion to classify AKI severity may be misleading. It was reported that the combination of creatinine and urinary output for classifying the patient's risk of death was more stringent than urinary output alone for classifying patients [7,24]. One can conclude that patients classified according to the urine output criterion only might be less severe than those classified according to the combination of creatinine and urine output [25]. On the other hand, severe tubular dysfunction can lead to increased urine output despite low GFR. Urine output therefore seems to be a nonspecific and poor parameter for classifying of AKI in critically ill patients.

## Glomerular filtration rate as a determinant of urine output

**Figure 1:**
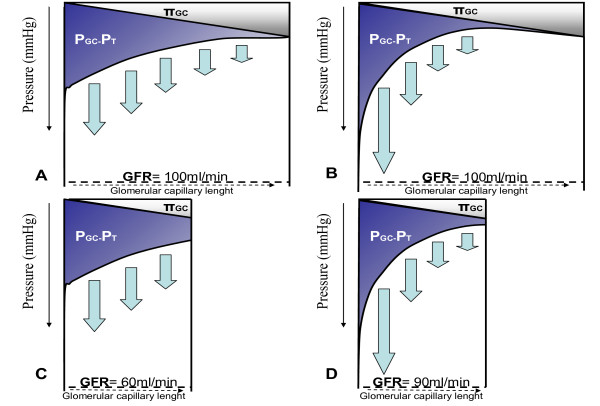
**Schematic representation of the glomerular capillary hydraulic and oncotic pressure in normal kidneys (A and B) and pathologic kidneys with decrease of the total ultrafiltration surface (C and D)**. The difference between the hydraulic pressure difference [P_GC_, glomerular capillary hydraulic pressure-P_T _hydraulic pressure in Bowman's space) and the intracapillary oncotic pressure (∏_GC_) represents the effective filtration pressure gradient. In normal condition (**A**), the P_GC_-_PT _slightly decreases along the glomerular capillary axe and the ∏_GC _increases leading to equilibrium between the opposing forces to filtration. If renal perfusion pressure and P_GC _increase (**B**), the point of equilibrium is reached earlier along the axe due to increase of filtration fraction. GFR does not change and only increase of renal plasma flow and decrease of filtration fraction causes the GFR to increase (**B**). GFR is likely to increase with rise of renal perfusion pressure if the filtration surface is impaired, the point of equilibrium not being reached (**C **and **D**). Note the role of plasma oncotic pressure. Infusion of crystalloid decreases plasma oncotic pressure due to hemodilution favoring the net filtration pressure while infusion of colloids increases plasma oncotic pressure therefore reducing GFR. GFR, glomerular filtration rate.

At constant hydraulic permeability of the glomerular filtration barrier, the glomerular filtration is driven by the pressure gradient across the glomerular capillary walls (Figure 1). The pressure gradient across the glomerular capillary wall is determined by the opposing forces of the hydraulic and oncotic pressures gradients between the capillaries and the Bowman's space. Because the length of the afferent and efferent arterioles in the glomerular capillary network is relatively short and the resistance is low, the glomerular capillary hydraulic pressure remains rather constant along the capillaries, whereas the oncotic pressure along the capillary increases in relation with filtration. Therefore, the limiting factors of GFR are the renal plasma flow and the plasma protein concentration. A higher renal plasma flow will induce a reduction in filtration fraction (i.e., ratio of ultrafiltration to renal plasma flow) with a lesser increase of capillary plasma protein concentration along the glomerular capillaries. Conversely, when the renal plasma flow is reduced, the glomerular filtration rate decreases but with an increase in the filtration fraction. An increase of capillary hydraulic pressure will cause the ultrafiltrate to be mainly generated on the first portion of the afferent side of the capillary network and to cease when hydraulic and oncotic pressures become equal along the glomerular capillary network (Figure 1). Therefore, the oncotic pressure becomes the limiting factor of glomerular filtration [26]. In this line, the natriuresis and diuresis response to crystalloids infusion are in part mediated by the changes of intraglomerular oncotic forces following plasma protein dilution [27,28], an effect that is not observed after hyperoncotic colloids administration. When hydraulic permeability is altered (decreased of glomerular surface area as in chronic kidney disease) glomerular hydraulic capillary pressure becomes the major determinant of the glomerular filtration rate (Figure 1) [29].

## Relationship between renal blood flow and GFR

Physiologically, the renal blood flow is autoregulated, which means that it remains unchanged when arterial blood pressure varies [30]. Such autoregulation is mediated by a myogenic mechanism, the tubuloglomerular feedback (TGF), and a "third mechanism" not yet fully identified. The lower autoregulatory threshold of mammalian kidney occurs at a mean arterial pressure (MAP) of ~80 mmHg. Below this pressure level, renal blood flow and glomerular filtration rate decrease along with the decrease in pressure [31].

In normal kidneys, the total interruption of renal blood flow for a prolonged period of time (i.e., more than 30 minutes) followed by reperfusion is always associated with major tubular and microvascular damage. In this condition, cellular lesions result from a combination of cellular hypoxia-reperfusion injury and oxidative stress-associated damage [32]. This situation is a rare clinical scenario except during suprarenal aortic surgery with aortic clamping. Experimental studies have shown that prolonged period of renal hypoperfusion would not systematically lead to renal histological damage and renal failure [33,34]. Saotome et al. reported that prolonged mechanical reduction of renal blood flow by 80% for 2 h in conscious sheep did not induce sustained renal function impairment or kidney damage [33]. In a rat model, Johannes et al. have shown that temporary mechanical reduction of renal blood flow does not impair microcirculatory oxygenation and renal function [34]. However, severe renal damage were observed in rats recovering from an ischemic acute renal failure induced by intra-arterial infusion of norepinephrine [35], which underwent additional injury by mild hemorrhage, an effect partially prevented by renal denervation. These observations highlight the role of renal innervation in the induction of renal failure. Together, these experiments suggest that a severe transient hypoperfusion is able to reduce GFR and urine output but is not sufficient to induce persistent AKI. However, this is the superimposition of renal hypoperfusion episodes in relation to other insults, such as sepsis or ischemia, which may induce renal failure. Because of the above-mentioned arguments, it is expected that preventing a decrease of renal blood flow may prevent or limit the occurrence of AKI in ICU patients.

Renal blood flow autoregulation exists at high mean arterial blood pressure, protecting the glomerular structure from hypertensive injury by a decrease of glomerular capillary pressure [36]. Therefore, one can expect that increasing renal perfusion pressure when MAP is below the threshold of renal blood flow autoregulation or if autoregulation is impaired could improve GFR and urine output through an increase of renal blood flow. Sepsis is the leading contributor to AKI in the ICU setting, accounting for more than 50% of episodes of AKI. Whereas fluid challenge can improve renal perfusion pressure and renal perfusion in hypovolemic states, the sole fluid resuscitation is unlikely to increase largely the mean arterial pressure. Vasopressor infusion is therefore required to improve renal perfusion pressure in conditions with systemic inflammation [37]. Norepinephrine has been reported to increase renal blood flow, urine output, and creatinine clearance in experimental sepsis [38]. Although norepinephrine also has been found to increase creatinine clearance in human sepsis [39], clinical studies in which MAP was increased with norepinephrine have provided conflicting results. Bourgoin et al. found that increasing MAP from 65 to 85 mmHg did not further improve creatinine clearance in patients with septic shock [40]. In contrast, in a more recent study among patients with vasodilatory shock after cardiac surgery, infusing norepinephrine was found to improve renal oxygen delivery, oxygen delivery/consumption balance, and GFR when MAP was increased from 60 to 75 mmHg [41]. Infusion of norepinephrine in septic patients titrated to increase MAP from 65 to 75 mmHg was associated with a decrease of renal Doppler resistive index, suggesting an increase in renal vascular conductance [42], confirming the experimental data. These results are in accordance with physiological animals studies that showed that norepinephrine and vasopressin can induce, in septic states, an increase of renal blood flow through a combined increase of renal perfusion pressure (i.e., prerenal mechanism) and an increase of renal vascular conductance (i.e., intrarenal mechanism) [38,43].

Such an increase of renal blood flow does not necessarily translate into GFR increase. For example, infusion of low-dose dopamine (2 μg/kg/min) can increase renal blood flow, induce renal vasodilatation, and increase urine output but with no effect on creatinine clearance [44].

**Figure 2:**
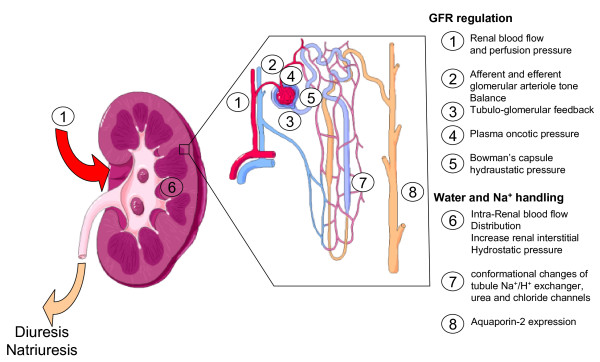
**Schematic view of regulating factors of diuresis and natriuresis**. Renal blood flow, renal perfusion pressure, and plasma oncotic pressure influence the effective filtration pressure gradient. Afferent and efferent glomerular arteriole tone can further influence the glomerular capillary hydraulic pressure while tubular cast accumulation increases the Bowman's hydrostatic pressure decreasing the effective filtration pressure gradient. Finally intrarenal blood flow distribution, conformational changes of tubule Na+/H+ exchanger, urea, and chloride channels and aquaporin-2 expression regulate water and sodium (Na^+^) handling of the ultrafiltrate (see text for further details). GFR, glomerular filtration rate.

These apparent conflicting findings call for several comments. First, increase of renal blood flow or urine output does not necessarily translate into increase of creatinine clearance. The systematic review of human AKI by Prowle et al. showed that renal plasma flow and GFR were poorly correlated [45]. In a septic hyperdynamic animal, a fall in creatinine clearance can occur despite an increase of renal blood flow [46]. The same group using the same model found that infusion of angiotensin II could improve creatinine clearance while depressing renal blood flow [47]. Ventilation with positive end expiratory pressure always decreases urine output in correlation with a decreased renal perfusion pressure (mean arterial blood pressure - renal venous pressure) and reduced renal blood flow [48]. A nonpharmacologic technique (lower body positive pressure) was used to increase cardiac output and renal blood flow but with no impact on diuresis [48]. In other words, increasing renal perfusion pressure can increase urine output and natriuresis independently of changes in total renal blood flow and GFR. These discrepancies could, in part, be due to the effect of neurohormonal regulation of vascular tone between the afferent and efferent glomerular arterioles (Figure 2). As an example, predominant vasodilatation on efferent arterioles leads to increase renal blood flow with a steady glomerular capillary pressure and GFR. Conversely, a predominant vasoconstriction of the efferent arterioles, even if renal blood flow remains unchanged, increases the GFR and urine output, potentially inducing renal ischemia. Second, renal fluid and sodium excretion (i.e., diuresis and natriuresis) can exhibit a pressure-dependency response [43,49,50]. Several humoral factors control sodium excretion through, in part, changes of renal medulla blood flow and intrarenal redistribution of blood flow.

## Role of intrarenal blood flow distribution in regulation of diuresis and natriuresis

Whereas normal kidneys receive ~20% of cardiac output, the medulla receives less than 10% of renal blood flow [51]. Even with a stable renal blood flow within the range of autoregulation, the cortical and medulla have different responses to changes in renal perfusion pressure (RPP). In contrast to the cortical microcirculation, the medulla microcirculation appears to be poorly autoregulated, i.e., pressure-dependent. Renal medulla blood flow regulation is of paramount importance with respect of the regulation of diuretics and natriuresis and, therefore, the response of the kidney to the body fluid composition and volume status (Figure 2). In fact, in mammalians kidneys, the ability of the medulla circulation to regulate its own blood flow depends largely on the body volume status. In euvolemic dogs, when a RPP is decreased from 153 to 114 mmHg within the range of RBF autoregulation (i.e., with no change of renal blood flow), flow in the inner medulla decreases with no redistribution of flow within the renal cortex [50]. In contrast, both renal cortical and medulla are well autoregulated in hydropenic rats. Because the descending vasa recta provide blood flow to the medulla emerge from efferent arterioles of juxtamedullary glomerules, these data suggest that changes in resistance in the postglomerular circulation of juxtamedullary nephrons might be responsible for the lack of autoregulation of medullary blood flow in volume expended animals [51]. Increase in renal medullary blood flow decreases the outer-inner medullar osmotic gradient and increases renal interstitial hydrostatic pressure, which both impair the ability to concentrate urine and participate in the natriuresis response to hypertension in well-hydrated mammalians. In hydropenic animals, this response is blunted preventing further loss of water and sodium. The tubular sodium handling may be mediated more by the angiotensin II and paracrine effects of NO rather than the increase in RPP *per se*. In the absence of angiotensin II, volume expansion with no increase in MAP induces natriuresis, whereas the increase in MAP by angiotensin II infusion did not induce a natriuresis response [52]. Increase of plasma vasopressin concentration (independently of any increase of systemic arterial pressure) also influences the pressure-natriuresis/diuresis relationship in decreasing the medullary blood flow through receptor V1a [43]. Binding to the V2-receptors in the inner medullary collecting ducts activates the UT-A1 molecules, which increases the urea permeability of collecting duct and increase the ability to concentrate urine. Increased vascular response of the renal microcirculation to vasoconstrictors has been proposed to elicit intense renal vasoconstriction in sepsis-induced AKI [53]. Although this hypothesis warrants further exploration, it is possible in sepsis that endogenous vasoconstrictors, including angiotensin II, could both decrease GFR due to decrease in renal blood flow but also blunt the natriuresis response after the renal perfusion pressure has been restored. Endotoxemia also can increase urine output and water clearance despite decrease in GFR due to tubular aquaporin-2 dysfunction [54].

The adaptation of medullary blood flow to the Na^+ ^concentration in the tubular lumen adds another level of complexity to the regulation of regional blood flow and sodium handling. The glomerular filtration rate will decrease due to vasoconstriction of the afferent glomerular arteriole in response to increase of the filtrated Na^+ ^reaching the macula densa, a mechanism called the tubuloglomerular feedback (TGF, Figure 2). Tubular salt sensing by the macula densa involves the Na^+^/K^+^/2Cl^- ^cotransporter (NKCC2). The mechanism of TGF consists in an increase of the glomerular afferent arteriole vascular tone, mainly mediated by adenosine release, in response to a raise of the [NaCl] concentrations in the tubular fluid. The juxtaglomerular apparatus also mediates renin-release signals through prostaglandins (i.e., PGI2 and PGE2) and nitric oxide release. The TGF response to increase of Na^+ ^concentration in the tubular fluid operates within a few seconds but is not sustained. Prolonged stimulation of the TGF will induce the TGF to reset within 30-60 minutes, increasing the renal blood flow without restoring the GFR [55]. Activation of the TGF has long been proposed by Thureau et al. as an adaptative mechanism to tubular dysfunction and referred as an "acute renal success" in acute renal failure [56]. In theory, TGF response could prevent the rapid loss of water and electrolytes in conditions of tubular dysfunction-associated decrease of Na^+ ^reabsorption. Na^+^-tubular reabsorptive work constitutes a major part of renal oxygen consumption in the healthy kidney. As a consequence, decrease of GFR or inhibition of Na^+ ^tubular reabsorption can decrease renal oxygen consumption [57]. However, in ischemic-induced AKI there is a diversion of oxygen consumption from Na^+ ^reabsorption to other oxygen-consuming pathways illustrated by an increase of the ratio oxygen consumption/Na^+ ^reabsorption [58]. Redfors et al. have recently shown in an elegant physiological study in patients developing AKI after cardiac surgery that total renal oxygen consumption increases despite a decrease of Na+ reabsorptive work [59]. The oxygen consumption to absorptive work mismatch is not well understood and may result from: 1) higher production of reactive oxygen species by infiltrative immune cells [60]; 2) high level of NO, which regulates the renal oxygen consumption [58]. This may partially explain why strategies designed to inhibit renal oxygen consumption (e.g., loops diuretics) have failed to improve the prognosis of patients suffering from AKI [61].

## Urine output, urine biochemistry, and mechanism of AKI

Medical textbooks provide urine biochemistry profiles to differentiate prerenal causes from intra renal causes of AKI in oliguric patients. Although very popular among clinicians, the ability of urinary indices, such as urinary Na^+ ^(UNa) and excretion fraction of Na^+ ^(FeNa), to separate prerenal from intrarenal causes of AKI is questionable. First, these urinary markers have been poorly studied among critically ill patients. Recent reviews of experimental and human sepsis have highlighted the paucity of available studies and their design heterogeneity regarding urinary findings in septic AKI [62,63]. Most importantly, there is no evidence that these urinary biochemical findings can predict the response to hemodynamic optimization in terms of renal injury and renal function. Although a low UNa or FeNa (e.g., FeNa <1%) suggest a preserved renal tubular reabsorptive capacity, there is no evidence for a correlation between urinary biochemical modifications and tissue damage. Inflammation mediators can induce tubular cell dysfunction with conformational changes of tubule Na^+^/H^+ ^exchanger, urea, or chloride channels that will influence urine composition independently of any structural damage [14,64,65]. As mentioned, the control of urinary Na^+ ^excretion results from a complex neurohumoral regulation and is influenced by fluid resuscitation, arterial pressure, or infusion of diuretics. A fractional excretion of urea (FeU) of 35% or less has been proposed to differentiate prerenal AKI from intrarenal causes independently of the use of diuretics. However, mechanically ventilated patients with transient AKI (resolving within 3 days) exhibited higher FeU than patients with persistent AKI in a recently published cohort [66]. To summarize, sensitivity and specificity of traditional urinary biochemicals showed significant disparities among clinical studies such that their value to classify AKI remains doubtful. There is much more expectation in the use of new biomarkers (i.e., NGAL, KIM1) to make an early diagnosis of tubular damage during the course of AKI and therefore to differentiate prerenal from intrarenal AKI in oliguric patients. Only a few studies are available regarding the association between plasma and/or urine levels of those biomarkers and the reversibility of AKI. Bagshaw et al. reported that plasma NGAL had an area under the ROC curve of 0.71 (95% confidence interval (CI), 0.55-0.88) for predicting AKI progression and of 0.78 (95% CI, 0.61-0.95) for need for renal replacement therapy. Cruz et al. reported an area under the ROC curve of 0.82 (95% CI, 0.7-0.95) for predicting the use of renal replacement therapy [67]. Nickolas et al. reported that urine NGAL remained low in patients admitted in the emergency department with prerenal azotemia versus AKI [68].

## Conclusions

Decrease urine output is common among critically ill patients and can mirror a decrease in creatinine clearance. Although a decrease in renal blood flow and/or a decrease in renal perfusion pressure is a major determinant of GFR, plasma oncotic pressure appears to be central in the glomerular hydrodynamic forces. In hypovolemic states, prompt fluid resuscitation is needed to prevent further deterioration of renal function. The choice of the type of fluid also seems to be crucial, because colloids increase the oncotic pressure and may reduce filtration rate. Fluid administration may be found inappropriate and even harmful in numerous situations due to the inconstant relationship between renal blood flow or renal perfusion pressure and diuresis/natriuresis due to complex neurohormonal control. Furthermore, systemic inflammation can induce natriuresis and diuresis changes due to functional changes unrelated to hypoperfusion, histological, or tubular damage. Experimental and clinical research is needed to determine appropriate therapeutic response to oliguria in critically ill patients.

## Competing interests

The authors declare that they have no competing interests.

## Authors' contributions

ML and DP wrote and approved the final manuscript.
